# Word sense disambiguation using hybrid swarm intelligence approach

**DOI:** 10.1371/journal.pone.0208695

**Published:** 2018-12-20

**Authors:** Wafaa AL-Saiagh, Sabrina Tiun, Ahmed AL-Saffar, Suryanti Awang, A. S. Al-khaleefa

**Affiliations:** 1 Knowledge Technology Research Group (KT), Centre for Artificial Intelligent (CAIT), Universiti Kebangsaan Malaysia (UKM), Bangi, Selangor, Malaysia; 2 Faculty of Computer System and Software Engineering, University Malaysia Pahang (UMP), Pahang, Malaysia; 3 Broadband and Networking (BBNET) Research Group, Faculty of Electronics and Computer Engineering, Universiti Teknikal Malaysia Melaka (UTeM), Hang Tuah Jaya, Durian Tunggal, Melaka, Malaysia; Universita degli Studi di Catania, ITALY

## Abstract

Word sense disambiguation (WSD) is the process of identifying an appropriate sense for an ambiguous word. With the complexity of human languages in which a single word could yield different meanings, WSD has been utilized by several domains of interests such as search engines and machine translations. The literature shows a vast number of techniques used for the process of WSD. Recently, researchers have focused on the use of meta-heuristic approaches to identify the best solutions that reflect the best sense. However, the application of meta-heuristic approaches remains limited and thus requires the efficient exploration and exploitation of the problem space. Hence, the current study aims to propose a hybrid meta-heuristic method that consists of particle swarm optimization (PSO) and simulated annealing to find the global best meaning of a given text. Different semantic measures have been utilized in this model as objective functions for the proposed hybrid PSO. These measures consist of JCN and extended Lesk methods, which are combined effectively in this work. The proposed method is tested using a three-benchmark dataset (SemCor 3.0, SensEval-2, and SensEval-3). Results show that the proposed method has superior performance in comparison with state-of-the-art approaches.

## 1.0 Introduction

The task of determining the meaning of words automatically in computational linguistics is reflected in word sense disambiguation (WSD). WSD has been a vital issue in natural language processing (NLP) for years, and it has been applied in various NLP tasks, such as information retrieval, machine translation, and automatic summarization. The most important clue for WSD is the context of an ambiguous word. Feature words are selected from the context to determine the right sense of the ambiguous word. Knowledge-based WSD usually selects words in a certain length of a window as feature words. Then, according to the relatedness between feature words and each sense of ambiguous word, the sense with maximum relatedness is selected as the right sense. Word ambiguity is categorized into polysemous and homonymy words. Polysemous words refer to words that have multiple senses with subtle differences, whereas homonymy words are words that have multiple senses, with each sense relating to a specific domain. Disambiguating homonymy words is easier than disambiguating polysemous words. Disambiguation requires an accurate quantifier that can measure the semantic relation between any two senses. Without such quantifier, a large annotated corpus, which is an expensive and time-consuming effort, is needed for the disambiguation process.

Several studies proposed different techniques for WSD. Such techniques are mainly based on annotated corpuses [1–3], semantic and syntactic approaches such as dictionary-based ones, and part-of-speech tagging and parsing [1, 4–7]. Recently, a new trend has emerged in the area of semantic disambiguation, in which WSD is treated as a combinatorial problem. Meta-heuristic algorithms are usually utilized for this problem [8–11]. Meta-heuristic algorithms in WSD search for the best combination of senses for a given text. An efficient optimization model leads the search process to the global optimum solution, which is represented by the global meaning of the given sentence. Optimization methods are based on either a population of solutions or a single solution. Population-based algorithms explore a wide area of a given search space. Thus, they are useful in diversification procedures. However, these types of algorithms do not adequately exploit the search space in comparison with single-solution algorithms. Establishing a balance between exploitation and exploration is necessary to reach the global optimum solution [12–17]. Hence, the current study aims to use an unsupervised model that exploits semantic relatedness and similarity methods to find the most suitable meanings of ambiguous words. This model is implemented using the swarm intelligence search algorithm. This algorithm maximizes the semantic relatedness and similarity depending on various methods that measure the aforementioned criteria. For the best of our knowledge PSO has not been implemented in the semantic disambiguation domain. However, it shows effective performance on similar domains such as part of speech tagging. This has motivated us to implement PSO for the semantic disambiguation where it has common task with POS-tagging problem.

The main factor in the semantic disambiguation is how the similarity or relatedness is measured. In this paper, the main contribution is to provide accurate evaluator that can estimate the semantic value for the inputted senses. This evaluator represents the core part of the proposed PSO, where it is used as fitness function of the algorithm. Also, we enforce the search process of the PSO by including local search algorithm. Incorporating such algorithm is frequently used to help the main search method in intensifying the search around a single solution. Using local search too often during the search process can lead to premature convergence. The combination of the two meta-heuristic approaches, namely, PSO (global search) and SA (local search) algorithms, increases the coverage of the proposed hybrid PSO. Meanwhile, the balance of exploitation and exploration leads to the global optimum of the search space and avoids the local minima [15]. Hence, we control the use of local search in each iteration by making it work within specific rate. Other methods may differ in this aspect by allowing the local search works iteratively or works based on a counter [18, 19].

We also proposed a novel combination of the extended Lesk (e-Lesk) algorithm and JCN method for all parts of speech (POSs). Our approach aims to combine the high-performance JCN for measuring the similarity of nouns and verbs and the e-Lesk algorithm that covers all POSs. The e-Lesk algorithm is considered as an effective measurement method for all POSs [20, 21].

This article is organized as follows. Section 2 presents the related studies in the field of WSD. Section 3 provides the proposed methodology involving language resource, semantic measures, and hybridized PSO and SA. Section 4 discusses the results of the proposed method, which is described and experimented with different window sizes. Section 5 analyzes the results of our model relative to other methods from the literature and our model limitations. Finally, Section 6 presents the conclusion of this work, along with final remarks.

## 2.0 Related work

This section reviews the related state-of-the-art WSD studies. The proposed WSD model falls in the meta-heuristic method. Hence, this section focuses on the search methods that use semantic similarity or relatedness.

Unsupervised approaches can avoid the knowledge acquisition bottleneck [22] [23], i.e., the lack of extensive resources that are manually labeled with word senses. Unsupervised approaches for WSD relies on the notion that the same sense of a word tends to have similar neighboring words. Here, word senses are driven through input text via the clustering of the occurrences of the word; then, new occurrences are classified into prompted clusters [24] [25]. These approaches are not reliant on labeled datasets, and they do not take advantage of any machine-readable resources, such as thesauri, dictionaries, or ontology. As long as these methods do not utilize any dictionary, they cannot rely on a shared reference inventory of senses; such shortcoming constitutes the main disadvantage of completely unsupervised systems [26, 27].

In the context of unsupervised WSD, a co-occurrence graph is a method that suggests a different view of word sense discrimination, and it has been recently explored with certain success [28]. The method is based on the concept of a co-occurrence graph, i.e., a graph G = (V, E) in which vertices V correspond to words in a text and edges E connect pairs of words co-occurring in a syntactic relation, in the same paragraph, or in a large context. This graph-based algorithm for large-scale WSD [29] [30] involves a few parameters and does not require sense-annotated data for training. This method examines several measures of graph connectivity to identify those best suited for WSD. Mihalcea proposed a graph-based algorithm for sequence data labeling by means of random walks on graphs encoding label dependencies [31] [32].

Intelligence search techniques are most similar to co-occurrence graphs. First, meta-heuristic approaches to solving the WSD problem has been successfully applied using the SA algorithm [33]. This SA method relies on the e-Lesk algorithm [34] to quantify the relatedness among words for the purpose of disambiguating these words.

In recent years, many meta-heuristic algorithms have been applied for WSD due to the success achieved in the reference [33]. These algorithms are based on a branch of solutions to explore additional solutions in the problem space. These types of algorithms have rapidly progressed in the domain of lexical disambiguation. The accuracy of population-based algorithms reaches a grade better than that of single-solution algorithms in WSD [33].

The genetic algorithm (GA) has been investigated for solving WSD [35, 36] [8, 37, 38]. GA maximizes the relatedness among words in sentences. In the method of Gelbukh et al. (2003), the relatedness measure is presented by the Lesk algorithm, which is based on the gloss overlap notion. Similarly, the ant colony system has been exploited to maximize the relatedness measured by the Lesk algorithm [39]. The Lesk overlap method cannot determine the physical length between two concepts (semantic similarity), especially between the concepts of nouns and verbs [40]. Hence, several studies have used the semantic similarity method proposed by Wu and Palmer [41] [42] in the maximization process of GA [36].

In the context of swarm intelligence methods, PSO has been successfully applied to NLP tasks [43]. For example, PSO hybridized with the k-means algorithm performs the process of document clustering [44, 45]. In this clustering method, the PSO algorithm initializes the centroid vectors of the clusters of k-means. PSO is also used for document clustering by integrating it with fuzzy k-means or other optimization algorithms [45–47]. The binary version of PSO is hybridized with the estimation of a distribution algorithm to achieve multi-document summarization [48, 49]. PSO is also applied in the domain of syntax disambiguation, i.e., POS tagging [50]. This method achieves high accuracy in disambiguation in two domains, namely, English language and Portuguese corpus. This advantage motivated us to apply PSO to WSD as WSD resembles the process of POS tagging.

## 3.0 Proposed technique

The model design of this study consists of four main phases.

Phase 1: This phase involves reading SemCor files and mapping fine-grained POS tags to coarse-grained POS tags.

Phase 2: This phase involves the use of hybrid PSO (i.e., PSO with SA) search, which is an efficient meta-heuristic algorithm, to maximize the entire semantic relatedness on a set of parsed words. This phase includes the following steps.

Step 1: This step presents the data. Each word in the sentence is mapped to a number that denotes the number of senses of the word being disambiguated.

Step 2: The fitness function that combines two semantic relatedness measures is implemented.

Step 3: The best solution among all pre-generated solutions is identified using the hybrid PSO search algorithm.

Phase 3: This phase involves the assessment of the quality of the final solution with regard to the benchmark corpus.

**Fig 1:**
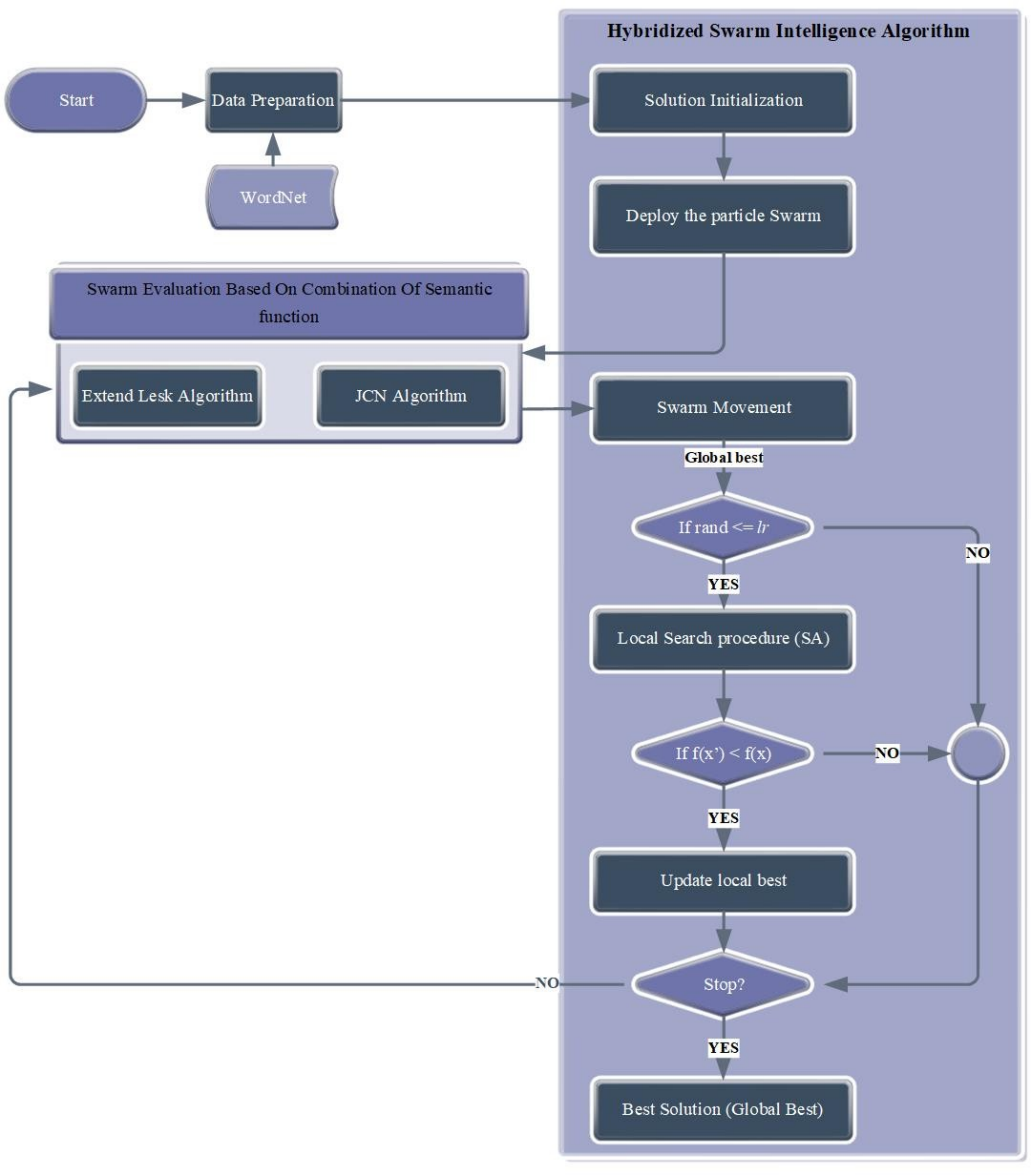
Proposed technique.

These phases are illustrated in Fig 1, and additional details are explained in the next subsections.

### 3.1 Language resource

WordNet is a common language resource that finds the taxonomic and networked relationships of English concepts, arranges these related concepts, and provides groups of synonyms that represent the concepts [51, 52]. All WordNet relations are a type of POS. The relation between two concepts is called hypernym when the first concept is a kind of the other concept, e.g., a car is a motor vehicle type. Meanwhile, the large hierarchy of noun concepts is an evident feature in a WordNet relation. These hierarchies of noun concepts, which comprise over 70% of the total relations for nouns, make up the distinguishing characteristic of WordNet. Similarly, the hierarchies for verbs, known as troponomy, are included; an example is walking being a troponomy of moving. The hierarchy of nouns and verbs can represent the tree. The general concept represents the root of the tree, and the specific types of this concept represent the leaves. In the current research, WordNet is used to provide the number of senses for each non-stop word. Moreover, the semantic measures used in this study rely on WordNet to provide the relations and glosses for each word of the processed text.

### 3.2 Semantic measures

Semantic measures can be categorized into two main classes, namely, semantic similarity and semantic relatedness. Semantic similarity indicates the dependencies among two concepts with respect to the related information of their hierarchy. Such measure can be accomplished by either the path length between two concepts in a hierarchy [53, 54] or the information content of the concept [55–57]. By contrast, semantic relatedness identifies the mutual relation between two concepts without considering POS tagging. An example of this measure is the Lesk algorithm [34, 58]. The present study utilizes a combination of JCN and the extended Lesk method to measure the similarity and relatedness of all POSs. This section presents three subsections to describe each semantic method used in this study and how they are employed to perform the fitness function.

#### JCN measurement

The JCN measurement [59, 60] method is designed to identify the similarity score between two concepts on the basis of the information content of the concepts’ senses. Eq 1 describes the mechanism of obtaining such score.

$${Sim}_{jcn}(C1,C2)=\left(IC(C1)+IC\;(C2)-2*IC\;(LCS\;(C1,C2))\right)$$ (1)

where IC is the information content of the concept and LCS is the lowest common subsume that subsumes the first concept (C1) and second concept (C2) in ontology. Information content is the measure of specificity of the concept that is computed as a negative log from the frequency of the concept to its root. The key characteristic of the JCN method lies in determining the similarity score between two POS tags. Such score is used in the fitness function of the proposed meta-heuristic approach. By using this method, the different tags are discarded in terms of the similarity score.

#### Extended Lesk gloss overlap [61]

One of the significant drawbacks of the JCN method is the discarding of the similarity score between two concepts with different POS tags. For this purpose, this study aims to utilize the extended Lesk gloss overlap as another measurement method. The key characteristic of gloss overlap methods is inspired by the idea of WordNet relations (has-part and is-a), which does not cover all possible relations between two concepts being measured.

The input of the extended Lesk algorithm comprises two concepts that need to be measured, and the output is a relatedness volume, which is shown as a numeric value. Eq 2 shows the formula proposed by Banerjee and Pedersen [61] for computing the relatedness between two concepts (C1 and C2).

$$\begin{array}{c} relatedness\left(C1,C2\right)=\sum score\left(R1\left(C1\right),R2\left(C2\right)\right)\\ \forall \left(R1,R2\right)\in RELPAIRS \end{array}$$ (2)

The Eq 2 implies the condition: If (R1, R2) as a pair of relation belongs to set of relation, then (R2, R1) must belongs to the same set.

#### Combination of JCN and e-Lesk method

The solution in this study is evaluated by assessing the semantic similarity and semantic relatedness for the required text. The objective function identifies the semantic coherence among every pair of senses (i.e., decision variables for the solution). The criterion for evaluating the solution is to quantify the semantic similarity and relatedness for the target text. In this work, the cost of the solution indicates the semantic coherence between each pair of senses (pair of decision variables in the solution). Hence, these methods work as an objective function for the proposed algorithm by finding the total semantic coherence of a sentence. For example, the extended Lesk algorithm measures the solution cost as follows:

$$\sum_{i=1}^{n}\sum_{j=1}^{WS}Gloss(S_{i})\cap (S_{j})$$ (3)

where n indicates the size of the processed text (solution dimensions) and WS is the window size of the measures.

The objective function is obtained by computing the quality of the created solutions. In this study, the extended Lesk algorithm finds the relatedness value of PSO solutions on the basis of Eq 3. Every pair of solution variables is measured using the extended Lesk algorithm, and the total value of all pairs is considered as the solution quality. Therefore, semantically coherent solutions provide a high fitness value. The extended Lesk algorithm is combined with the JCN method for nouns and verbs as JCN does not yield a high value for unrelated concepts. Although the extended Lesk algorithm finds certain overlaps of unrelated senses, with the first senses of the given words being unrelated, it still produces similarities. Hence, to reduce this distortion, we include the JCN method as follows:

$$\sum_{i=1}^{n}\sum_{j=1}^{WS}log(Gloss(S_{i})\cap gloss(S_{j})+(IC(S_{i})+IC(S_{j})-2*IC(LCS(S_{i},S_{j}))$$ (4)

Where IC represents the information content for the given sentence and LCS is the lowest common subsumer. The logarithm of the gloss overlap is computed to converge the value of the extended Lesk to the value of the JCN as the latter computes similarity on the basis of information content. Otherwise, the JCN method would not affect the measurement as it is smaller in scale in comparison with the extended overlapping method.

### 3.3 Hybrid PSO

The proposed method employs two types of meta-heuristic search algorithms, with each type being characterized by special search ability. This section explains the mechanism of each algorithm and how they work together for achieving a high-quality search method.

#### Particle swarm optimization (PSO)

**Fig 2:**
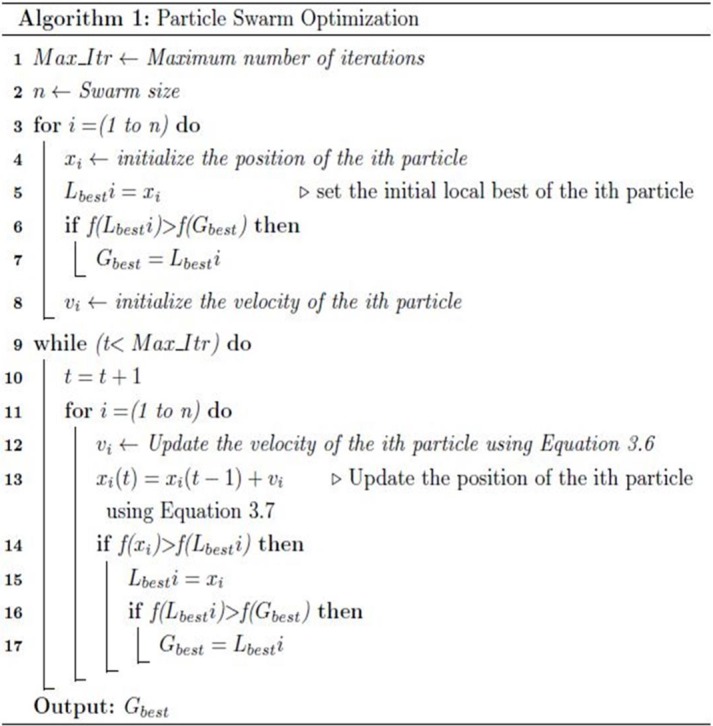
PSO pseudocode.

PSO was first developed by Kennedy and Eberhart (1995) and is based on the swarm intelligence manner that mimics birds flocking in nature. Potential solutions in PSO, termed as particles, fly over the problem space by going after the two best values, as shown in Eqs 5 and 6. The two best values are called *pbest* and *gbest*, which denote the best solution achieved and the best value gained by any particle in the population, respectively. All particles have objective values that are obtained via the objective function to be improved. They also have velocities in the direction of their inertia. Hence, the best objective value and best location are induced from the population at the end of the search.

$$v_{j}(t+1)=w\times v_{j}(t)+c_{1}\times rand()\times \left(x_{pbest,j}-x_{j}(t)\right)+c_{2}\times rand()\times \left(x_{gbest}-x_{j}(t)\right)$$ (5)

$$x_{j}(t+1)=x_{j}(t)+v_{j}(t+1)$$ (6)

where j = 1,…,n is the index of the jth element in the swarm, v represents the particle velocity, c1 and c2 are learning factors, w is the inertia weight that balances between global and local exploration, and rand() is a random number between 0 and 1. The velocity equation is used iteratively to update the previous velocity, as shown in line 12 of Fig 2. The second equation represents the solution (x) movement of the jth swarm, which is used to update the position of the particle iteratively, as shown in line 13 of Fig 2.

In WSD, each word has a specific number of senses with regard to the used lexical database. A word in a given sentence corresponds to a solution variable in PSO. Thus, each variable in the solution has different boundaries. The disambiguation process begins with the deployment of random guesses for each sentence related to variable limits, as shown in lines 3–8 of Fig 2. Then, the proposed guesses are improved iteratively with regard to the global and local best values, as shown in Fig 2.

#### Simulated annealing (SA)

**Fig 3:**
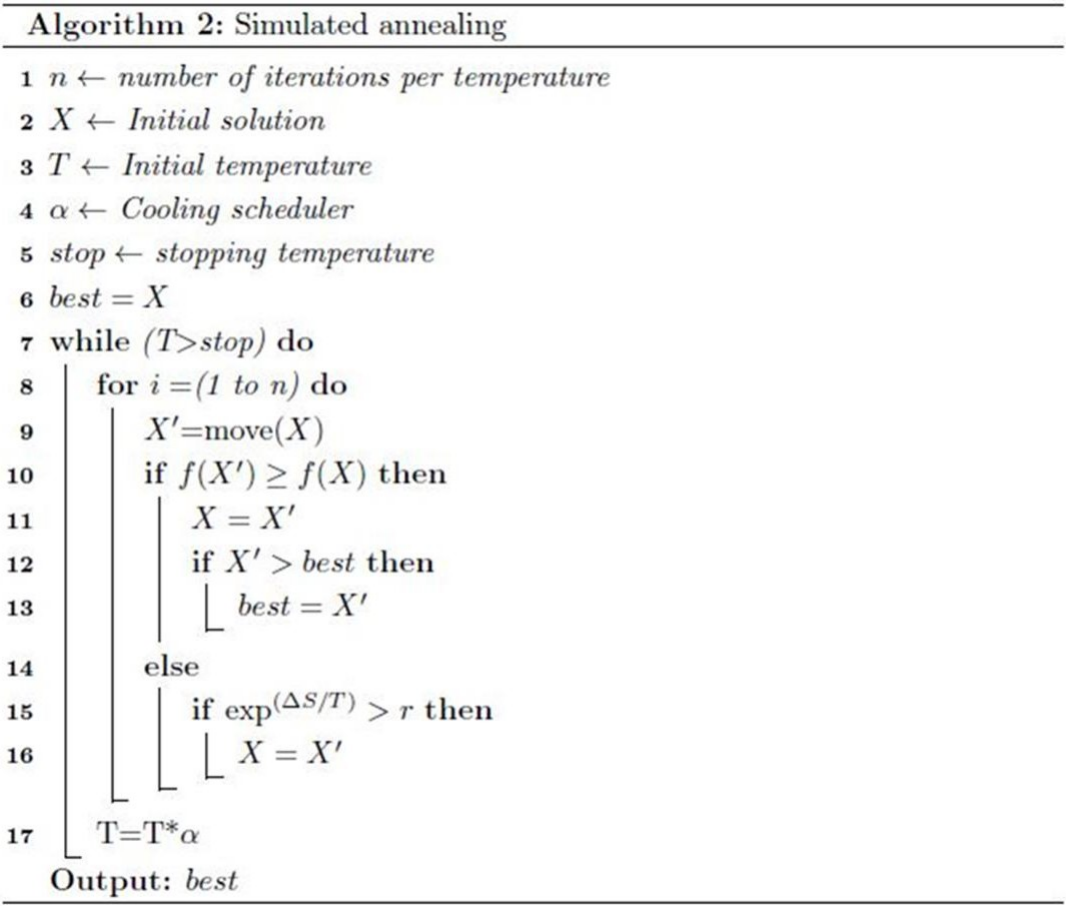
Pseudocode of SA algorithm.

SA is a meta-heuristic search algorithm that mimics metal cooling operations [62]. This algorithm searches intensively around a single potential solution by looking for improved neighbor solutions. This algorithm begins with an initial solution and improves it iteratively (Fig 3). In each iteration, the solution is moved to the neighbor solution according to the neighborhood structure, as shown in line 9 of Fig 3. The procedure of the movement in this study is based on a swap operation. However, after swapping, the solution must be tested for feasibility as each variable of the solution has specific boundaries (number of senses). Thereafter, a new solution is accepted if its semantic value is better than that of the previous solution, as shown in line 11 of Fig 3. The solution can also be accepted if it is not better than the previous one, but the new solution should satisfy the following condition:

exp(ΔS/T)>R (7)

SA interprets slow cooling as a slow decrease in the probability of accepting poor solutions. Hence, the above condition relies on the temperature value (T) to accept a poor solution. Meanwhile, *ΔS* denotes the difference between the current and new solution costs.

#### Hybrid PSO for WSD

This study hybridized a population-based algorithm called PSO with a local search algorithm called SA algorithm. This hybridization aims to achieve an accurate search algorithm that can identify a set of appropriate senses for ambiguous words in a given text. SA incorporates PSO algorithm to intensify the search process for a candidate solution.

**Fig 4:**
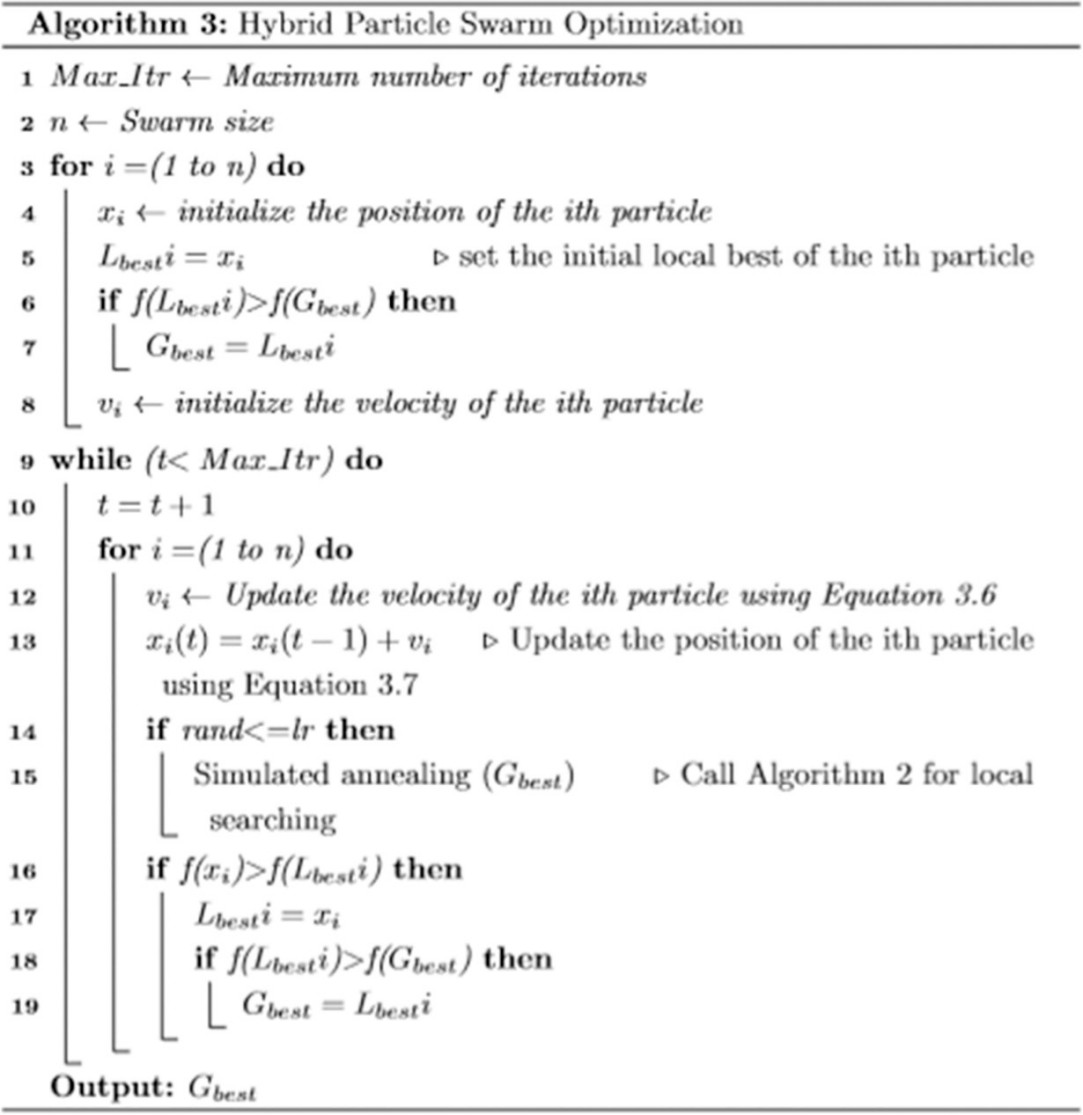
Pseudocode of hybrid PSO.

In our proposed method, the global best of the PSO is passed to SA to search the space around this solution (Fig 4). The local search is executed within a specific rate to maintain population diversity. Fig 3 shows that the local search is controlled by a condition consisting of a local search rate. This rate is determined experimentally in our study as 0.2; large values can lead to premature convergence.

## 4.0 Experimental results

The experimental results of the proposed method are based on the standard metrics shown in Table 1. These results are discussed in this section. The impact of the local search is shown through the experimented window sizes.

**Table 1:** WSD evaluation criteria.

Metric	Formula	Description
Coverage	$Coverage=\frac{\mathrm{all}\;\mathrm{answered}\;\mathrm{senses}}{\mathrm{total}\;\mathrm{of}\;\mathrm{senses}}$	It is the ratio of all answered senses to the total of the possible senses.
Precision	$\mathrm{Pr}ecision=\frac{\mathrm{correctly}\;\mathrm{answered}\;\mathrm{senses}}{\mathrm{total}\;\mathrm{of}\;\mathrm{answered}\;\mathrm{senses}}$	It is the ratio of the correctly answered senses to the total of the answered senses.
Recall	Recall=correctlyansweredsensestotalofsenses	It is the ratio of the correctly answered senses to the total of the senses.
F-measure	Fmeasure=2Recall*PrecisionRecall+Precsion	It is the harmonic average of precision and recall metrics.

### 4.1 WSD corpus processing

The evaluation of the designed model in WSD is based on specific data that reflect the accuracy of the proposed model. Two types of datasets are used to evaluate WSD systems. The datasets designed to examine the methods for one-word targets consist of a number of contexts that include a single word to be disambiguated.

Machine learning approaches target the aforementioned data. However, the proposed model is designed to tackle ambiguous words in a given text. Hence, this method is examined using other types of datasets. These datasets should consist of sentences that require disambiguation. Thus, each word in a sentence, excluding the stopping words, is ambiguous. Therefore, the designed model must assign the appropriate sense for each word. Moreover, the examined model must consider all possible senses for each word.

The designed model is experimented on the basis of the semantic concordance (SemCor) corpus, which is part of the Brown corpus [63]. Such corpus was labeled using WordNet 1.6 senses in the reference [64]. This corpus consists of a set of files that cover 234,000 word occurrences. The SemCor corpus is composed of 500 files, of which 186 files are annotated with senses for all occurrences of verbs, nouns, and adjectives. Each file in this dataset consists of sentences that contain words that are associated with POS tagging, word stem, number of senses, and orthographic form of the word. From this dataset, we use 19 files that have been used in related works. These files are br-a01, b13, c0l, d02, e22, r05, g14, h21, j0l, k01, k11, l09, m02, n05, p07, r04, r06, r08, and r09. The SemCor corpus is free and available online at web.eecs.umich.edu/ *mihalcea/downloads.html (see S1 Fig).

Utilizing a predefined library is vital in the SemCor file given its unique structure. For this purpose, we use the Java library of JSemCor to retrieve the line contents of SemCor sentences. The Java library of JSemCor is free and available online at https://projects.csail.mit.edu/jwi/. This library provides wide usage functions that can extract each part of a line and return it separately. For instance, it returns POS, lemma, and number of senses separately (see S2 Fig).

### 4.2 Semantic measures based on window of words

The context of a vague word in WSD is the main key to solve the ambiguity of that word. Window size refers to certain selected words that surround an ambiguous word; these words are used in the later stages of the disambiguation process. In the current study, the proposed model uses the window of words as a context identifier. The size of this window affects the disambiguation task by adding semantic information to the processed words. Hence, different window sizes are considered in this work, and the one with the best size is selected. To show an example of window of words, we quote a sentence from the dataset.

“Nothing in English has been ridiculed as much as the ambiguous *use* of the words, unless it be the ambiguous use of sentences.*”*

In the given example, the italicized word is the target ambiguous word, whereas the underlined words are the window context used to disambiguate the target word. This example shows a window of three words, including the target word. Certain words are neglected during the selection of the window as they are stop words (e.g., “of” and” the”).

Different window sizes are used in the study to show the impact of wide and narrow windows. The results of the semantic measures for each window size are shown in Tables 2 and 3. The results of the combined measures are presented in Table 4.

**Table 2:** Empirical results of e-Lesk for three different window sizes.

**Size**	**POS**	**Coverage**	**Precision**	**Recall**	**F-measure**
**Three words**	Noun	100%	66.93%	66.93%	66.93%
	Verb	75.26%	45.72%	34.41%	39.26%
	Adjective	100%	73.29%	73.29%	73.29%
	Adverb	100%	63.14%	63.14%	63.14%
	All	93.01%	64.25%	59.75%	61.92%
**Five words**	Noun	100%	67.69%	67.69%	67.69%
	Verb	75.26%	46.91%	35.30%	40.28%
	Adjective	100%	73.39%	73.39%	73.39%
	Adverb	100%	63.88%	63.88%	63.88%
	All	93.01%	65.11%	60.55%	62.75%
**Eleven words**	Noun	100%	70.20%	70.20%	70.20%
	Verb	75.26%	48.52%	36.50%	41.66%
	Adjective	100%	73.68%	73.68%	73.68%
	Adverb	100%	64.41%	64.41%	64.41%
	All	93.01%	66.19%	61.55%	63.78%

**Table 3:** Empirical results of JCN measure for three different window sizes.

**Size**	**POS**	**Coverage**	**Precision**	**Recall**	**F-measure**
**Three words**	Noun	100%	69.72%	69.72%	69.72%
	Verb	75.26%	47.98%	36.20%	41.26%
**Five words**	Noun	100%	72.63%	72.63%	72.63%
	Verb	75.26%	50.28%	37.84%	43.19%
**Eleven words**	Noun	100%	72.96%	72.96%	72.96%
	Verb	75.26%	50.81%	38.24%	43.63%

**Table 4:** Experimental results of the combined measure for an 11-word window size.

POS	Coverage	Precision	Recall	F-measure
**Noun**	100%	73. 36%	73. 36%	73. 36%
**Verb**	75.26%	51.16%	38.50%	43.93%
**Adjective**	100%	73.80%	73.80%	73.80%
**Adverb**	100%	64.76%	64.76%	64.76%
**All**	93.01%	67.44%	62.73%	64.99%

The increase in window size shows a gradual improvement in the results (Table 2). This improvement is due to the enrichment of the semantic information gained from widening the window of the words. However, expanding the window context slows down the system because of the need for additional semantic measures. Moreover, increasing the window size excessively may negatively affect the accuracy of the system, and noisy words are included in the semantic measures. The study of Lu et al. (2012) revealed that the window size of 16 words and above has an undesirable impact on the disambiguation of target words.

Table 3 shows the disambiguation results of nouns and verbs only as JCN cannot measure the semantic relation of other POSs. The reason behind this limitation is the structure of WordNet hierarchies, which serve as the foundation of JCN. No connection among different hierarchies exists in WordNet. Thus, JCN can only measure the concepts that belong to one hierarchy. In comparison with the results gained by e-Lesk, JCN shows higher accuracy for nouns and verbs. However, JCN is limited to only these POSs. Hence, our model in this research combines JCN and e-Lesk to perform the objective function of the hybrid search method.

The results of the final system are given in Table 4, which shows the disambiguation results based on the hybrid PSO and combined measure. Improvement is observed in the two measures when they work independently. Table 4 shows the results for the window size of 11 words only as the search method generally performs well with wide windows, as seen in Tables 2 and 3.

### 4.3 Hybrid PSO for WSD

The experimental results in the previous subsection are based on PSO using different types of objective functions, i.e., extended Lesk, JCN, and combined measures. The main goal in WSD is to find a suitable meaning for an ambiguous word on the basis of its context. Hence, the objective function of PSO is a semantic measure that provides a numeric value of the processed text. This section shows the impact of the local search algorithm on the process of finding the best possible combination of senses for targeted text.

PSO enables the global search of the problem space to find various solutions of different qualities. A local search algorithm, on the contrary, intensifies the search and processes a promising solution to be improved by searching its neighborhood. The local search method used in this model is the SA algorithm. This algorithm accepts unsatisfactory moves to break out of the local optima in the problem space of WSD. However, accepting non-improving moves is limited by a stochastic condition that considers the SA parameter called temperature. exp(*ΔS*/*T*), where ΔS is the difference between the new solution and the previous solution, T is the current temperature, and r is a random number between 0 and 1. Table 5 shows the SA parameters and their values used in this study.

**Table 5:** SA parameters.

Parameter	Name	value
T	Temperature	1
N	Epoch length	20
α	Scheduling factor	0.99
stop	Stopping temperature	1e-8

**Fig 5:**
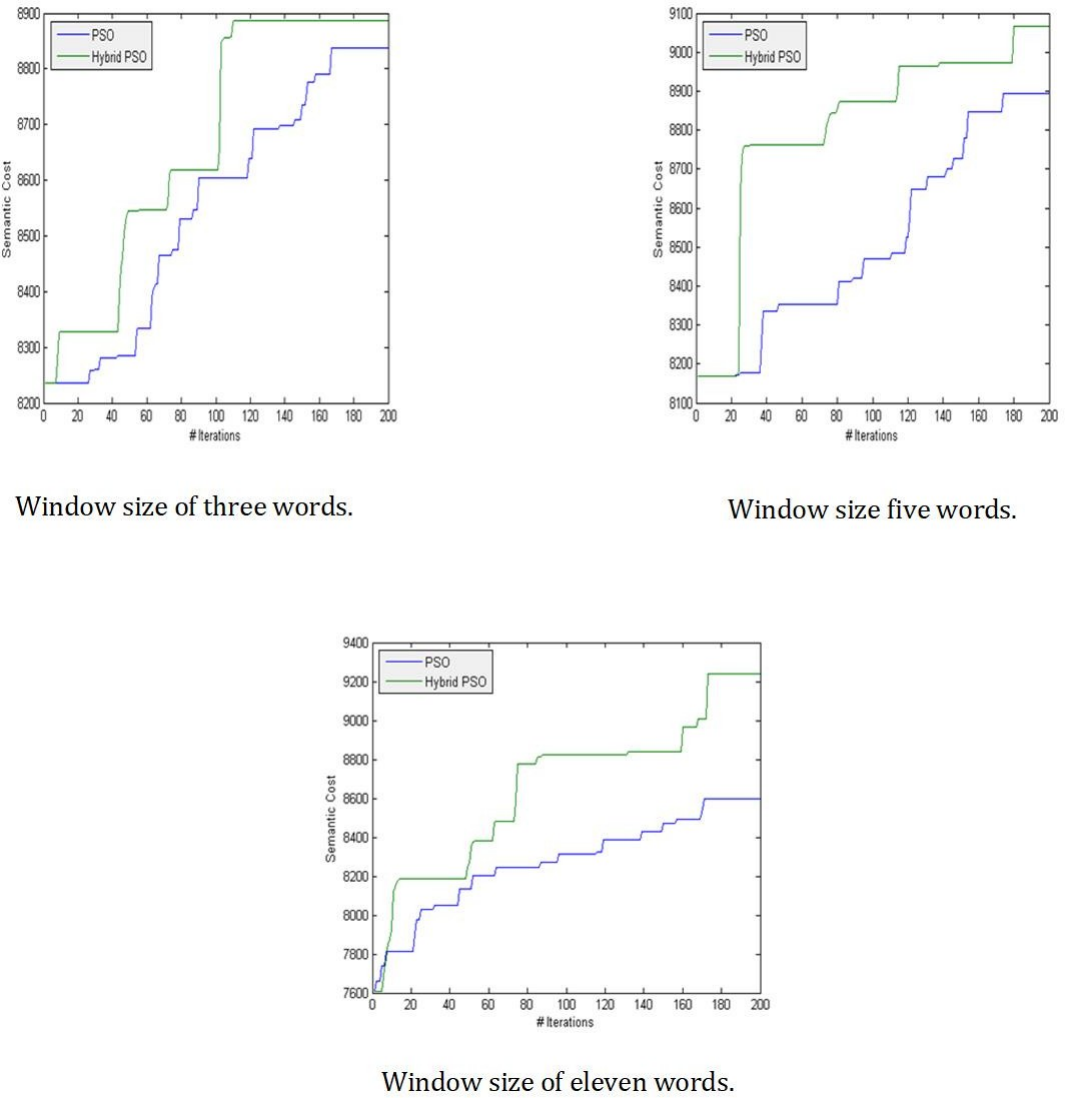
Hybridization impact on the search process of various window sizes.

In this study, the local search algorithm works at a specific rate to gain diverse PSO solutions. Thus, the problem space can be explored and exploited effectively. Fig 5 shows the impact of the local search algorithm on the search process.

Fig 5 shows the effect of the local search algorithm on the search procedure. The green line represents the hybrid PSO, i.e., PSO with SA, and the blue line shows the search results of PSO only.

The proposed search aims to find the maximum semantic relatedness among the words of processed text. Hence, the vertical axis in Fig 5 represents the semantic cost that increases gradually with the number of iterations in the horizontal axis. The pattern of lines in Fig 5 shows that the hybrid PSO produces considerable semantic relations, especially for large window sizes. As for small window sizes, the cost of PSO is close to that of the hybrid PSO. Thus, using a local search exerts considerable effects in a broad context.

## 5.0 Discussion

**Fig 6:**
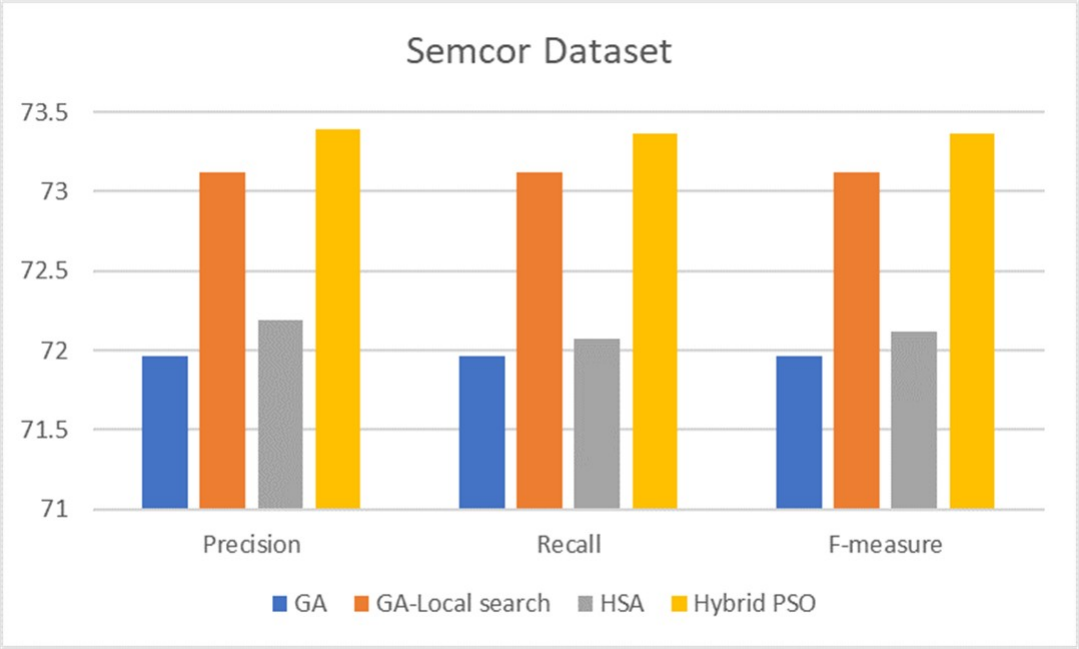
Comparison of results of hybrid PSO and related work based on SemCor 3.0 nouns POSs.

**Fig 7:**
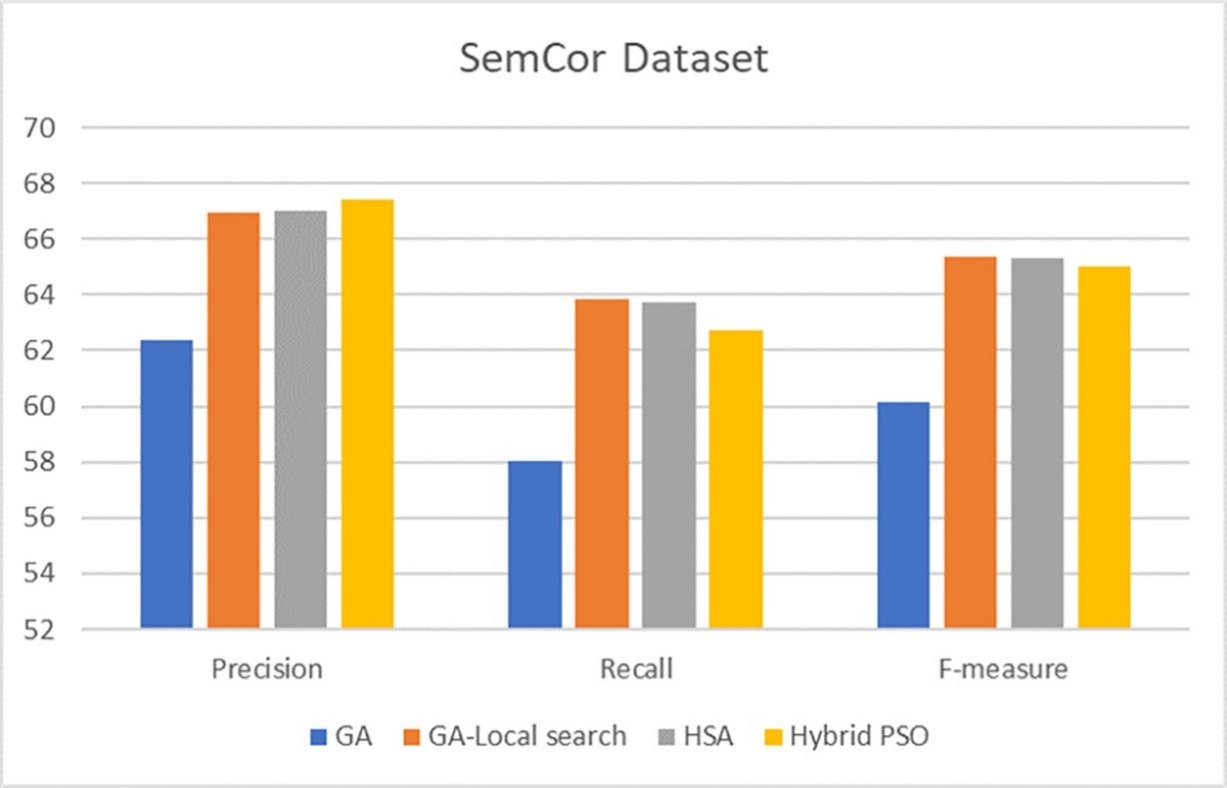
Comparison of results of hybrid PSO and related works based on SemCor 3.0 all POSs.

**Fig 8:**
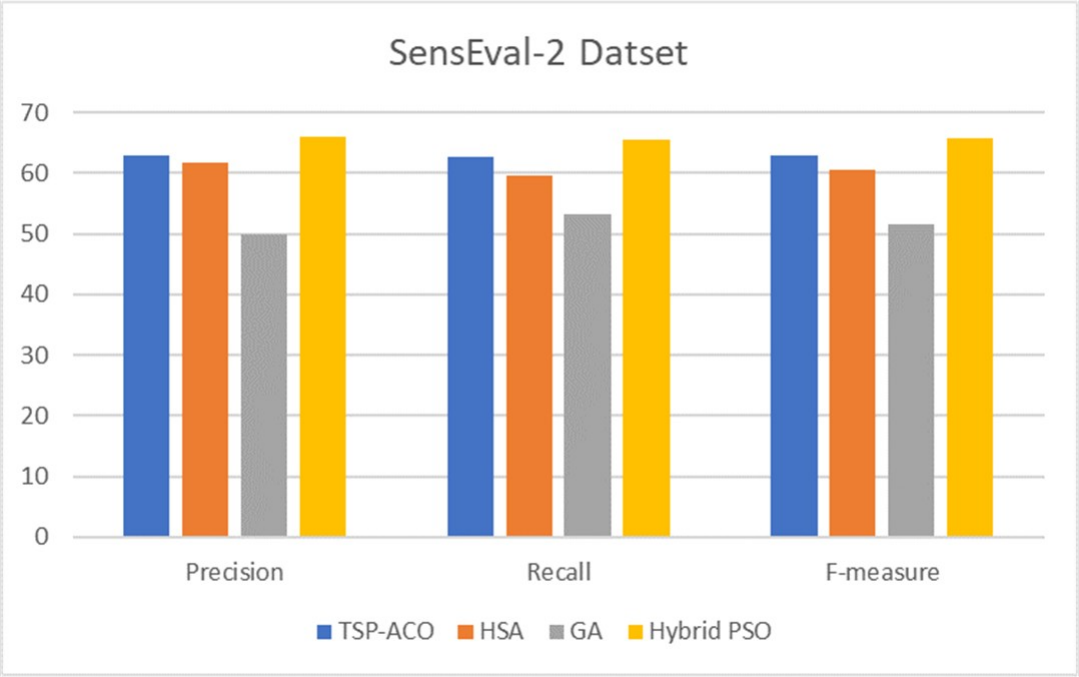
Comparison of results of hybrid PSO and related works based on SensEval-2 POSs corpus.

**Fig 9:**
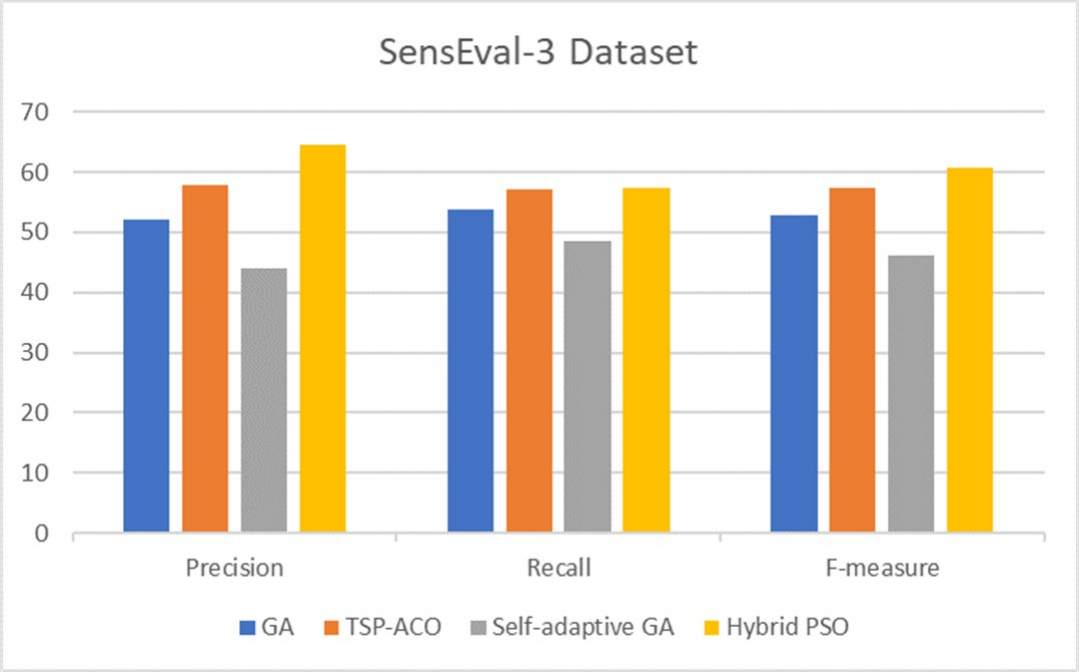
Comparison of results of hybrid PSO and related works based on SensEval-3 all POSs corpus.

To show the effectiveness of the proposed technique, we reported a comparison of three corpora based on all POSs (Table 6). We selected related works that use similar search methods, i.e., meta-heuristic algorithms. Then, we discussed the results of our proposed technique with the related works using nouns (Fig 6). In addition, we presented a comparison of the results of the corpora (Figs 7–9), along with a corresponding analysis and discussion according to all POSs.

**Table 6:** Comparison of results of hybrid PSO with the related works of three corpora using all POSs.

	Technique	Precision	Recall	F-measure
**Semcor 3.0**	Genetic algorithm GA [37]	62.38	58.07	60.15
	GA-Local search [8]	66.97	63.85	65.37
	Harmony Search Algorithm (HAS) [9]	67.03	63.73	65.34
	**Our proposed technique (Hybrid-PSO)**	**67.44**	**62.73**	**65.00**
**SensEval-2**	Traveling Salesman Problem using Ant Colony Optimization (TSP-ACO) [39]	63.00	62.80	62.90
	Harmony Search Algorithm (HAS) [9]	61.70	59.72	60.69
	self-adaptive GA [38]	49.82	53.27	51.49
	**Our proposed technique (Hybrid-PSO)**	**66.04**	**65.62**	**65.83**
**SensEval-3**	genetic algorithm GA [37]	52.13	53.79	52.95
	Traveling Salesman Problem using Ant Colony Optimization [39]	57.80	57.20	57.50
	Self-adaptive GA [38]	43.95	48.59	46.15
	**Our proposed technique (Hybrid-PSO)**	**64.68**	**57.43**	**60.84**

Fig 6 shows a comparison of the proposed method and two different GAs [8, 36] and the performance of the proposed method relative to the harmony search algorithm (HSA) [27]. The precision of the compared methods varies, with 71.96% being the lowest value of the genetic-Local search algorithm and with 73.36% being the highest precision of the hybrid PSO. The other metrics for the hybrid PSO are higher than those of the other methods as the hybrid PSO has 100% coverage for nouns. Nevertheless, HSA and GA-Local search have higher accuracy than the hybrid PSO with regard to verbs. Furthermore, these methods have high coverage, thus obtaining high recall and F-measure values. These methods attain F-measure values of 50.42% and 50.66% for the HSA and GA-Local search, respectively. The hybrid PSO in our model gained 43.93% for the F-measure of verbs. The hybrid PSO outperforms the other methods when all POSs are considered. Fig 7 shows a comparison of the hybrid PSO and other methods based on all POSs.

The GA in Fig 7 was proposed in the study of Hausman [28]. The comparison shows the superior performance of the hybrid PSO in terms of precision. In terms of recall, the proposed model in this work is not as accurate as HSA and GA-Local search. This result is due to the coverage of the hybrid PSO, which is not as large as that of the other methods. Therefore, the proposed model competes well with state-of-the-art meta-heuristic WSD.

Fig 8 shows the above results for the hybrid PSO on the SensEval-2 corpora [65] in terms of all POSs compared with the three investigated related works. In the method of [39], the travelling salesman problem using ant colony optimization (TSP–ACO) is applied to maximize the similarity. TSP–ACO obtains the best F-measure of 62.90%, which is lower than the value in the current work for the same corpora (SensEval-2). Moreover, Abed [9] reported an F-measure accuracy of 60.69% using (HSA), whereas our technique obtained improved accuracy at 65.83%. In practice, Abed [9] had been using Stanford Parser as a parsing method to gain additional grammatical relation information whereas our method gains better accuracy without the parsing phase. In addition, the new version of GA, namely, self-adaptive GA, [38] obtains only 51.49% accuracy. On the other hand, our hybrid PSO obtains 14.34% improvement in comparison with the self-adaptive GA [38]. By contrast, our proposed technique outperforms the other approaches that use TSP–ACO, HSA, and self-adaptive GA.

The results presented in Fig 9 show a comparative analysis of the hybrid PSO and related methods applied to the SensEval-3 corpora [66]. Compared with previous methods, hybrid PSO outperforms the other techniques [37], [39], and [38] in terms of precision, recall, and F-measure. Meanwhile, the study of [37] and [38] implemented two versions of GA for WSD. Those studies achieved overall F-measures of 52.95% and 46.15%, respectively. The method proposed in [39] outperforms the other related measures using the TSP–ACO technique. The performance of TSP–ACO reveals an F-measure of 57.50%, which is obtained by the phase of the graph centrality scheme. However, our proposed technique obtains better results at 60.84% without using any graph centrality scheme. For more results information on each POSs based SensEval-2 and SensEval-3 corpora (see S3 Fig).

The accuracy of the combined search method depends mainly on the semantic measure. This type of measure does not provides precise accuracy. Hence, the combination between local search and global search in this paper provides balanced search that can yield the best accuracy regarding the used semantic measure. However, other methods incorporate language resources or domain knowledge tagged corpus during the measurement process to enforce the semantic measure [39, 67, 68]. This enforcement makes the senses evaluation exploit much time. In this paper, we restricted our method to use the standard sematic measure, where this study focuses on the semantic optimization problem rather than knowledge-based disambiguation. Hence, we compared our method to the similar methods that used standard semantic similarity and relatedness measures.

## 6.0 Conclusion

This research utilized a meta-heuristic approach of PSO to identify the best solution (i.e., sense). The proposed PSO utilizes a local search algorithm using SA to improve the search of a neighborhood. In addition, we investigated the effect of window size on the disambiguation process. Hence, we presented experimental results for each window size to highlight their impact on our model. We also proposed a novel combination of semantic similarity and relatedness methods. The results of these methods and of the final model were presented independently. The final results of our model were shown in comparison with the results of related studies. This comparison was based on the same metrics applied on a three-benchmark dataset (SemCor 3.0, SensEval-2, and SensEval-3). Certain related works have presented their results on the basis of nouns only. Thus, we compared our results on the basis of this POS. Our experiments in the SemCor 3.0 dataset showed that the F-measure of the hybrid PSO is close to the best results of the related work, whereas our technique yielded the highest precision of 67.44% in terms of all POSs. Our proposed technique significantly outperforms other state-of-the-art techniques implemented with SensEval-2 and SensEval-3 datasets based on all POSs. Experimental results show that our novel combination of semantic measures along with the meta-heuristic hybrid PSO achieves the best results for varying datasets. In addition, the proposed method effectively improves WSD in comparison with other meta-heuristics approaches.

## Supporting information

S1 Fig Sample of Semcor’s dataset. (TIF)

S2 Fig Retrieve the SemCor Sentences line contents by Java library of JSemCor. (TIF)

S3 Fig Comparison of results of hybrid PSO based on SensEval-2 and SensEval-3 corpora of each POSs. (TIF)
