# Supplementary figures and images for: Word sense disambiguation using hybrid swarm intelligence approach
Source: PLoS One. 2018 Dec 20;13(12):e0208695. doi: 10.1371/journal.pone.0208695 (PMC6301655; doi:10.1371/journal.pone.0208695)

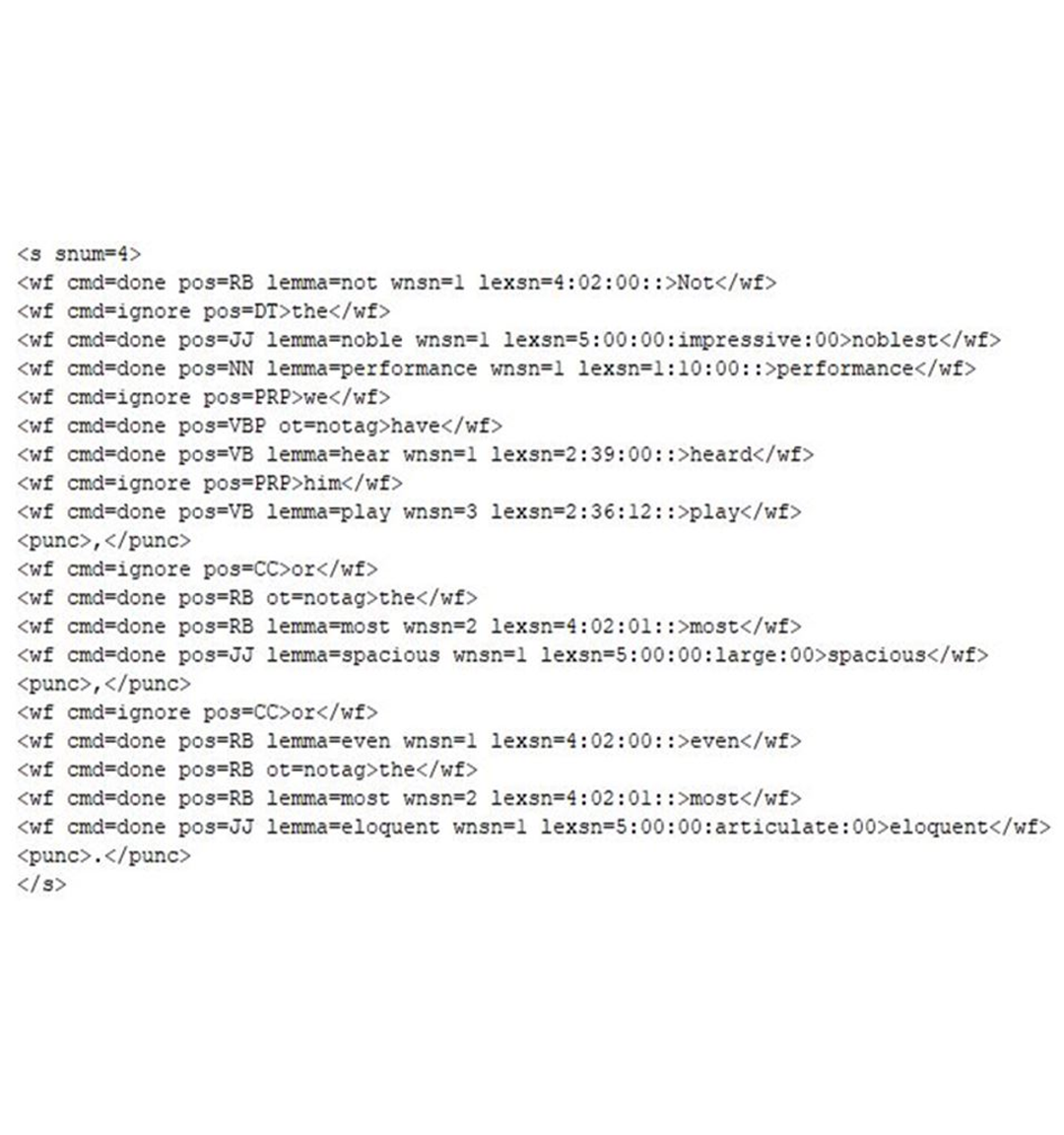

Supplement: S1 Fig — (TIF) [file pone.0208695.s001.tif]

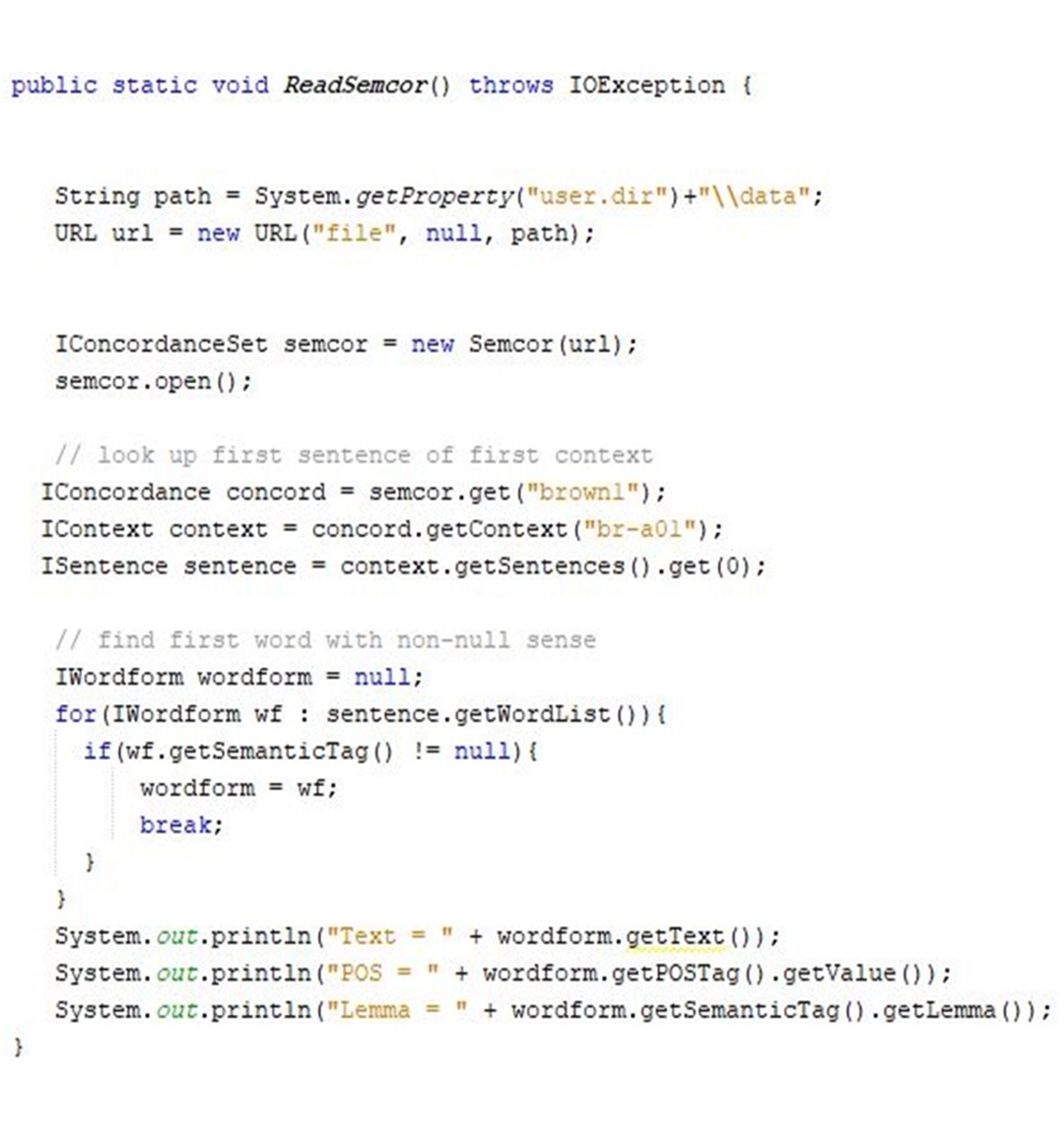

Supplement: S2 Fig — (TIF) [file pone.0208695.s002.tif]

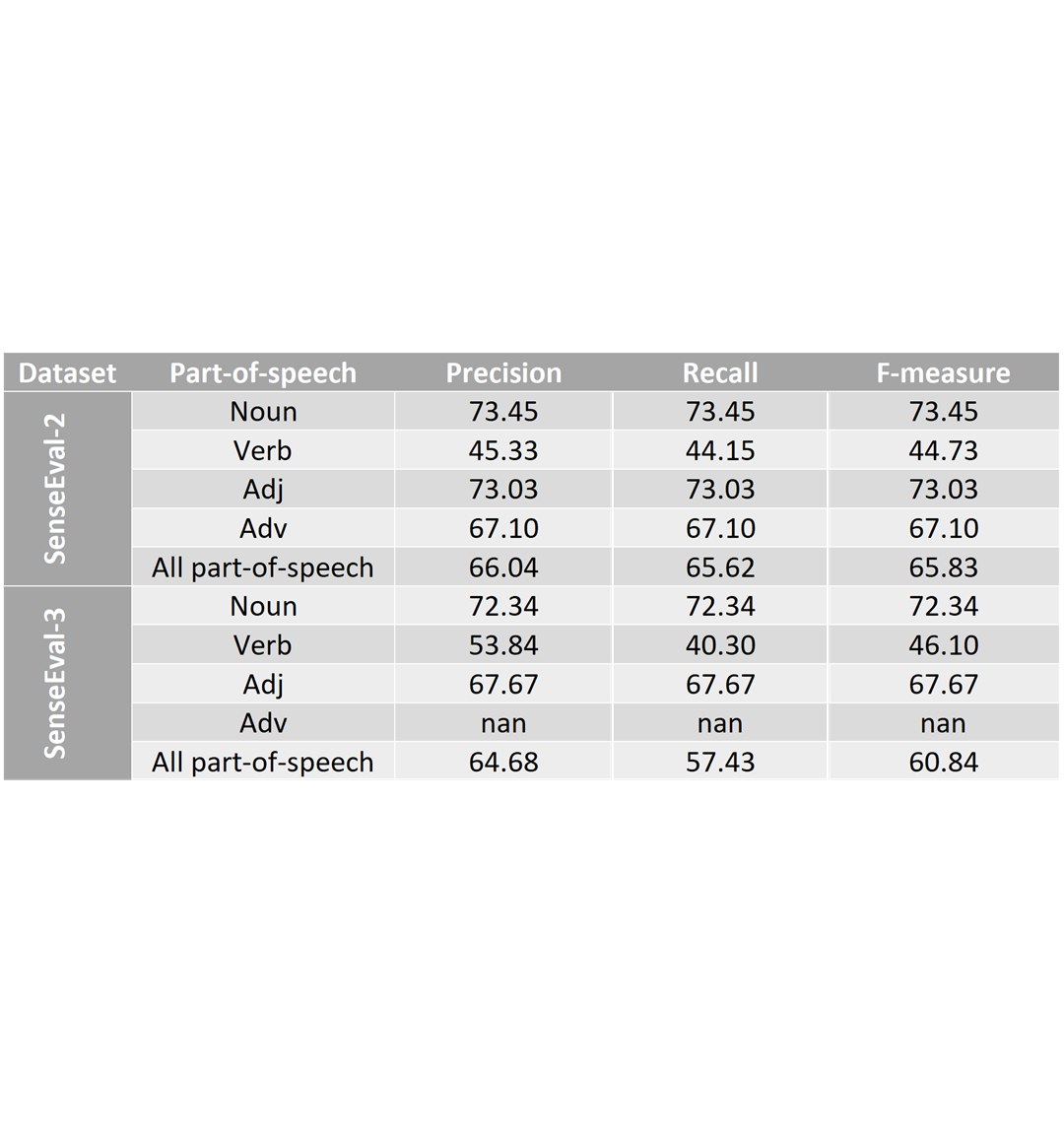

Supplement: S3 Fig — (TIF) [file pone.0208695.s003.tif]
